# Ceramic Inlays: A Case Report

**DOI:** 10.7759/cureus.29043

**Published:** 2022-09-11

**Authors:** Swapnil Pawar, Vikas Lekhwani, Hina Ahmed, Pooja Agrawal, Prajakta Barapatre, Pragati Sharma, Saurabh Gupta, Bharat Gupta

**Affiliations:** 1 Department of Endodontics, Yogita Dental College, Khed, IND; 2 Department of Prosthodontics and Crown and Bridge, People's College Of Dental Sciences, Bhopal, IND; 3 Department of Dentistry, Ibn Sina National College for Medical Studies, Jeddah, SAU; 4 Department of Prosthodontics and Crown and Bridge, New Horizon Dental College and Research Institute, Bilaspur, IND; 5 Department of Prosthodontics, Mahatma Gandhi Dental College and Hospital, Jaipur, IND; 6 Department of Prosthodontics, Bhagwatii Dental Care Hospital, Gorakhpur, IND; 7 Department of Endodontics, D.A.V Centenary Dental College, Yamuna Nagar, IND; 8 Department of Periodontology, MGM Dental College, Navi Mumbai, IND

**Keywords:** endodontics, esthetics, cast inlay, ceramic, inlay

## Abstract

Aesthetic dentistry continues to evolve via advances in bonding agents, restorative materials, and conservative preparation methods. Alternatives to dental amalgam and gold include ceramic dental restorative materials. The lifespan of ceramic inlay repairs is still up for debate. When it comes to durability, colour matching, and anatomical shape stability, ceramic inlay restorations top the list of options. More predictable long-term performance may be achieved by strategically placing ceramic inlays in teeth that are not subjected to significant occlusal stress. Preparation design for ceramic inlay materials is necessary to avoid flexure. This case report discusses the ceramic inlay practice for functional and aesthetic restoration in a patient.

## Introduction

Class I and II metal repairs may now be replaced with ceramic inlays for improved aesthetics. Repaired posterior teeth with intact buccal and lingual walls are the major targets of these devices. With these restorations, the buccal and lingual walls of the back teeth are left intact, allowing for tooth structure to be preserved while also using the mechanical advantages of contemporary adhesive technology to reinforce the damaged tooth. Ceramic inlays are a good alternative to amalgam or cast gold restorations, which have been shown to work in clinical trials [1].

Ceramic inlays have shown better physical qualities than posterior composite resin restorations, and when preparation margins are located in enamel, they may minimize microleakage more than either amalgam or gold. When the cervical margin is placed in the dentin, existing adhesive techniques have not totally prevented microleakage [2]. Ceramic inlay restorations must also be able to adapt to their surroundings in order to minimize resin cement deterioration and plaque build-up. Resin cement wear is caused by exposing the resin cement to the oral environment via a small crack at the repair. Drift and food can be irritants, which can lead to secondary caries and pulp devitalization [3] when there are only small differences. 

## Case presentation

**Figure 1:**
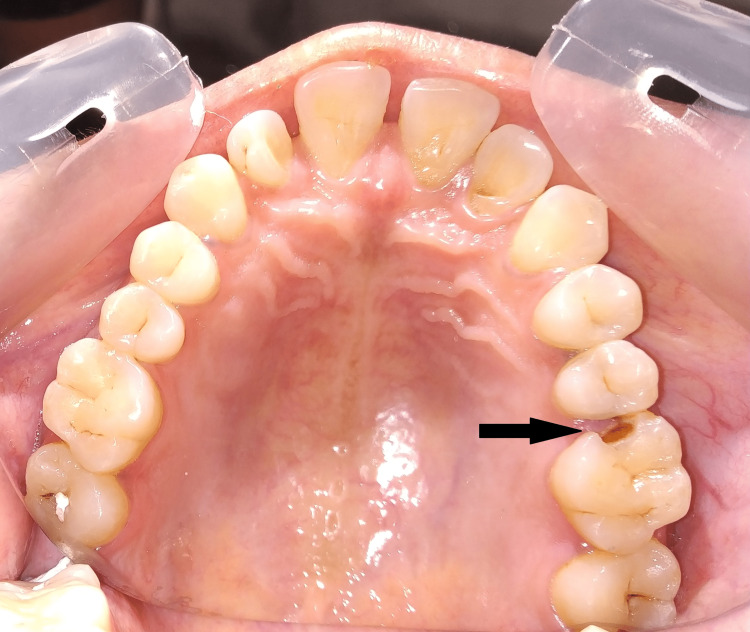
Preoperative intraoral view

**Figure 2:**
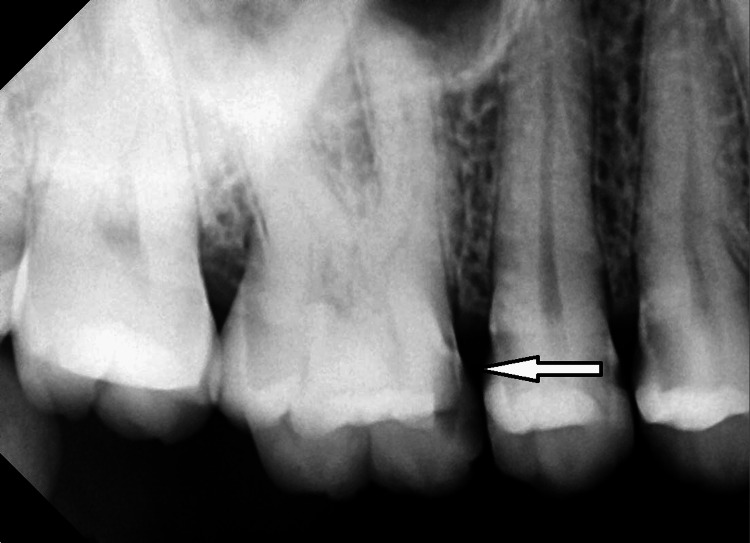
Radiographic image preoperative

A 28-year-old female patient presented to the department of endodontics and conservative dentistry with a complaint of food lodgement in the upper left back teeth region. After clinical and radiographic examination, we found distoproximal caries with 25 and mesio-proximal caries with 26 (Figures 1-2).

**Figure 3:**
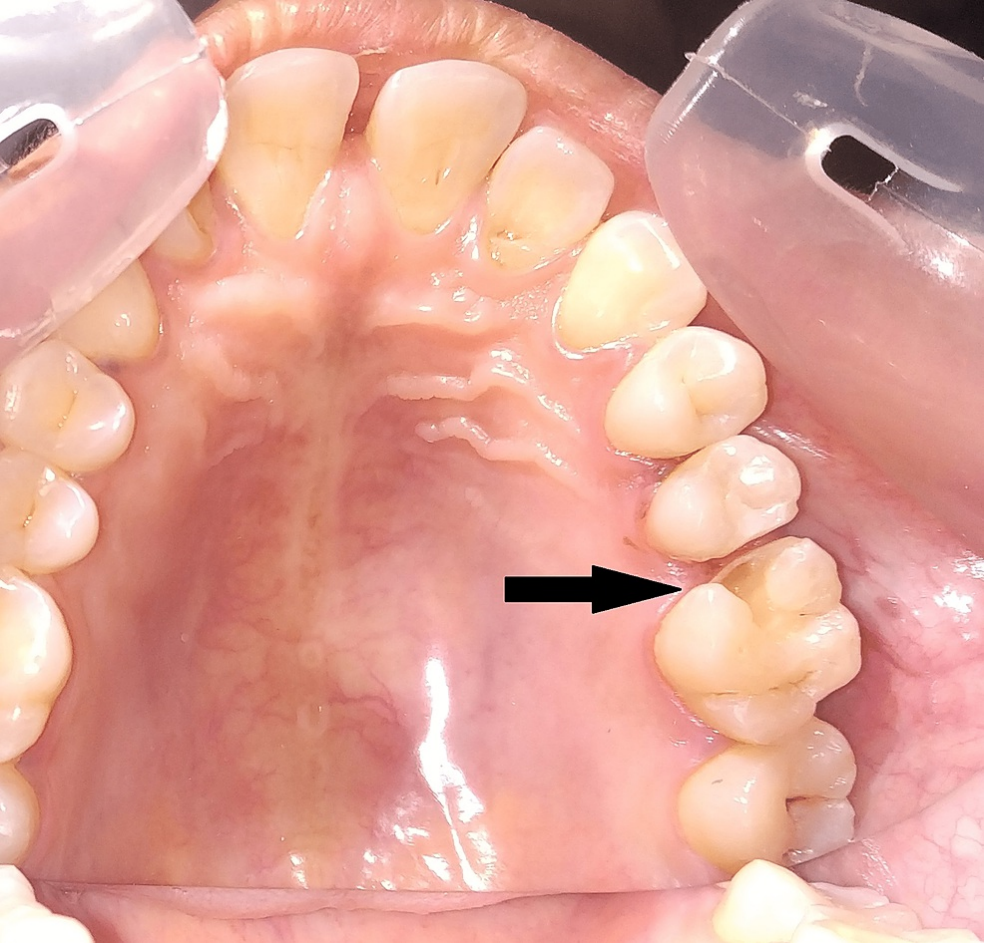
Cavity preparation

After the examination, a treatment plan was made including all the restorative treatment options which were explained to the patient. Since the patient requested aesthetic restoration, we decided on composite restoration with 25 and all ceramic inlay restoration with 26. Caries were excavated and restored with Filtek Z250 (3M ESPE, St. Paul, MN, USA) composite restoration with 25, The cavity was prepared according to the principles of inlay cavity preparation with 26 (Figure 3).

**Figure 4:**
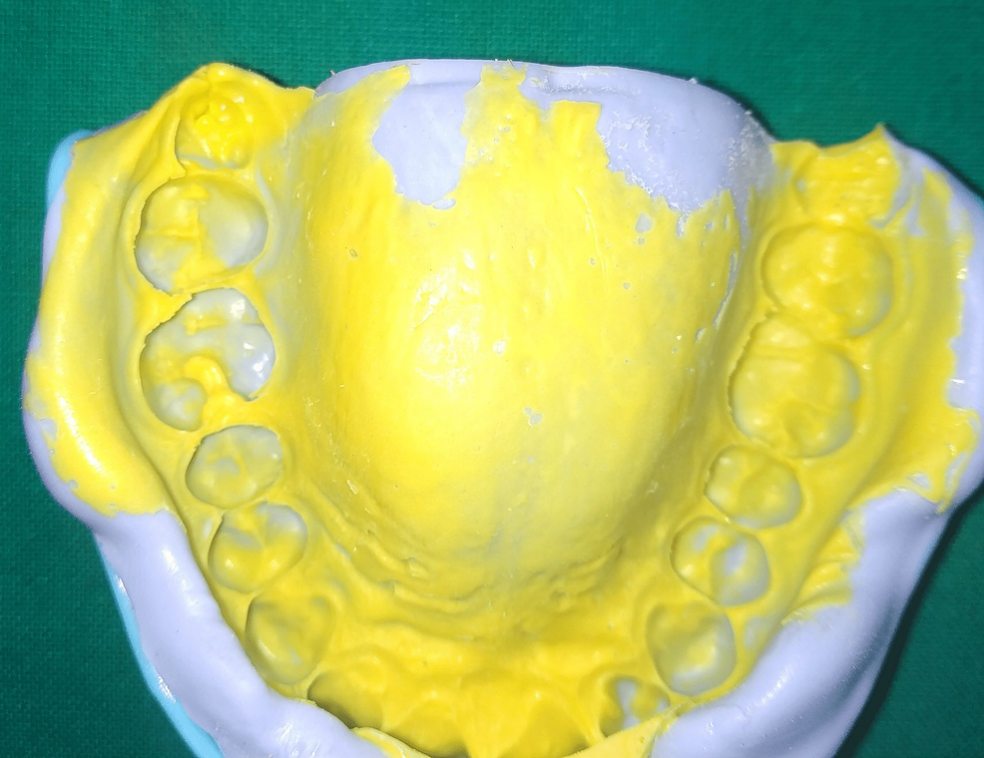
Impression of the preparation

After cavity preparation, cavity margins were examined and impressions were made with Dentsply Aquasil (Dentsply Sirona, India) and send to the laboratory for ceramic inlay fabrication (Figure 4).

**Figure 5:**
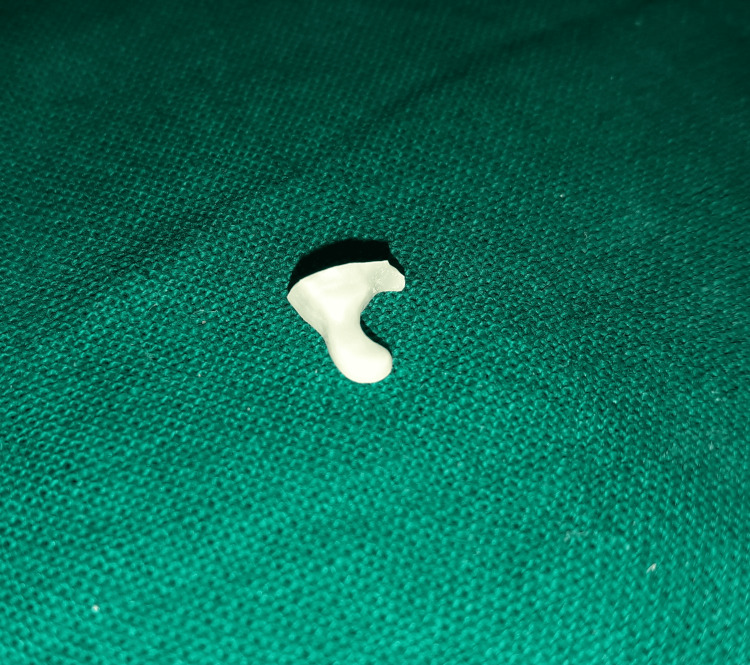
Ceramic cast inlay prepared from labaratory

In the laboratory, image was scanned from the cast and dye cut was prepared, and milling of ceramic inlay was done with CEREC Zirconia block (Dentsply Sirona, India) (Figure 5).

**Figure 6:**
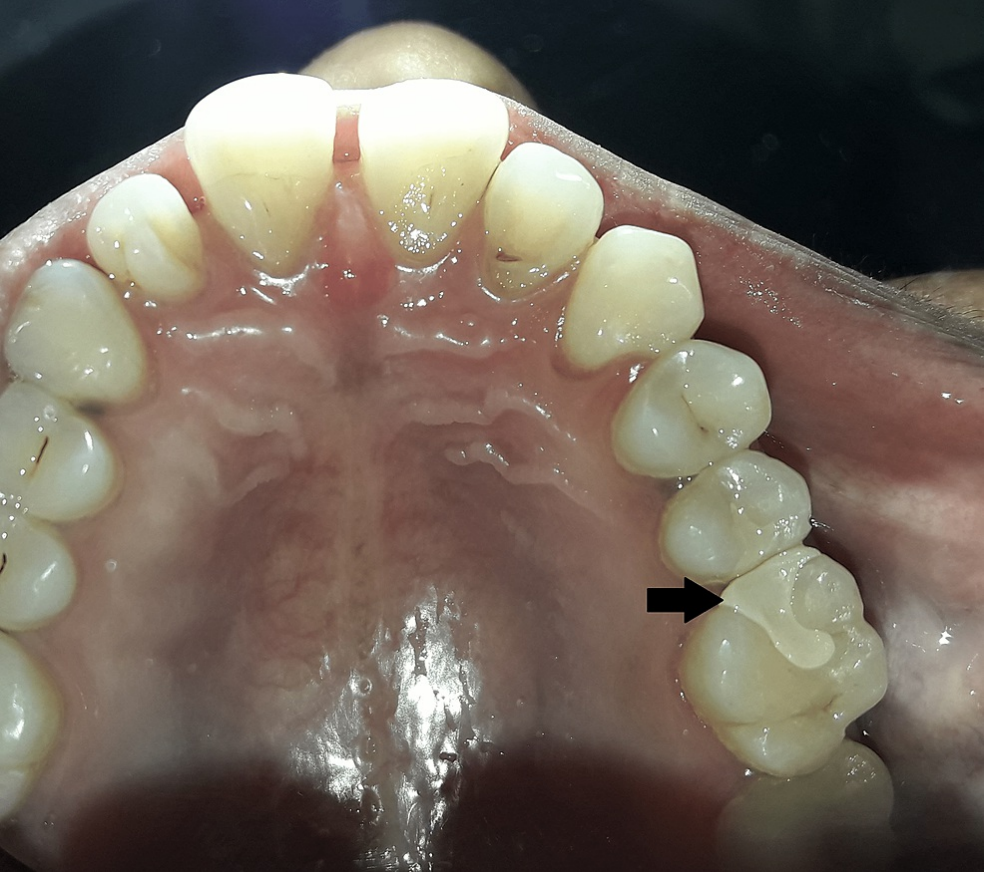
Postoperative view after cementation of inlay

After fabrication of inlay, it was checked for its polishing and marginal adaptation and it was etched with 9.5% hydrofluoric acid, silanted using silane coupling agent, and cemented using RelyX U200 self-adhesive universal resin cement (3M ESPE, St. Paul, MN, USA) (Figure 6).

After cementation, the inlay was checked for its marginal adaptation. The patient was recalled after seven days for evaluation of sensitivity, pain, and marginal adaptation and no such findings were seen.

## Discussion

Because of the extraordinarily large holes, it is possible that using direct back composite reclamation will not be possible. In this particular instance, aberrant rebuilding efforts were used for tooth reconstruction because they were superior to direct reclamations in a variety of ways. High biocompatibility, the inability of dental plaque to stick to their surfaces, and the fact that clay is stronger than composite tars and gives better results [4] are some of these ways.

In comparison to the outstanding cast trim, the underlying small variation of computer-aided design/computer-aided manufacture (CAD/CAM) reclamations, also known as CEREC, was quite poor; however, it has significantly improved as a result of recent improvements. The minor and inner hole sizes may have an effect on the life duration of the luting specialist as well as wear, staining, spilling, debasement, and the ability of the reconstruction to withstand stacking. Analyses of the trim and hole sizes after luting reveal very minor differences, with the range covering 136 to 278 microns. Despite advances in processing technology, the problem of overmilling is still a topic of concern for a significant number of people. In any case, the relatively large inner hole might make it possible for these reclamations to be located further away, thereby cutting down on the minor hole widths [1].

The point of intersection of all pit walls should be at 900, the cavo-surface point should be adjusted to 0.5 millimetres from nearby teeth, and the floor and gingival sheet region should be level and smooth. These are some of the most important aspects of pit design. Other important aspects include: the cavo-surface point should be adjusted to 0.5 millimetres from nearby teeth. In addition, the way in which all pit walls are drawn ought to be the same, presuming that there are undercuts that can be blocked with glass-ionomer concrete [5].

Ceramic inlays are an excellent choice to consider if you would prefer a less radical change to the appearance of your teeth during the design process. If a patient requires a class II restoration, in which both the buccal and lingual walls of the mouth are left unharmed, blend or composite rebuildings can be used instead of metal-projecting ones. They can also be used as a viable alternative in situations where the use of an immediate back composite rebuild would be impossible due to the extreme width of the isthmus in question. Clay trims outperform direct-back composite saps in terms of real characteristics because direct-back composite saps can only go through a limited amount of polymerization change [6].

Ceramic inlays have a greater risk of shattering when traditional corrosive-based reaction concretes are used, such as zinc phosphate and glass ionomer. This is in comparison to adhesively reinforced fired inlays, which are less likely to break [7]. The gum-altered glass ionomer luting experts gained a considerable amount of notoriety as a result of their practically unmatched strength in comparison to regular glass ionomers and the anticipated arrival of fluoride. This allowed them to surpass the strength of regular glass ionomers [8]. Given how quickly fluoride dissolves in tar-altered ionomers and how soft they are when compared to composite gums, it is hard to know what this last option means in terms of clinical use [9].

To achieve regions of strength for a bond with hydrofluoric corrosive-carved clay, it is possible to use a low-thickness glue gum at the gum rebuilding connection point to achieve the desired results [10]. The utilisation of silane coupling specialists results in an even more significant improvement to the bond. These specialists enhance the wettability of the clay through the sap and better organize the compound bonds [11]. When a proper technique is used, such as attaching a clay trim to a carved finish, the amount of microleakage that occurs is significantly reduced. Depending on the material used, a hydrofluoric corrosive drawing of clay, followed by the use of an expert in silane coupling, the bond that was made could change [12].

The lack of recurrent canes, the slight changes in marginal discoloration and color match, combined with excellent longevity was demonstrated in the clinical investigation by Fuzzi and Rappelli which proves that ceramic inlays are a valuable tool for the restoration of posterior teeth [13]. According to the findings of a study conducted by Manhart et al., the placement of posterior tooth-colored inlays by relatively unskilled student operators resulted in a success rate of 100% for ceramic inlays and 90% for composite inlays [14]. Lange's clinical trial showed that indirectly-produced Evopress (Wegold Edelmetalle AG, Wendelstein, Germany) ceramic inlays worked better than directly-produced Filtek Z250 composite restorations in terms of marginal adaptation, color match, and anatomic shape [15].

There has been more than one study that compares the flexure that is displayed by ceramic inlays to that of composite inlays in terms of the stresses that are placed on them. These findings, which are based on a finite-element model, suggest that teeth that have been restored with composite inlays are more flexible than teeth that have ceramic inlays. If the dentin-bonding agent interface is stressed more in composites with a low modulus of elasticity, this suggests that porcelain inlays are less likely to come loose than composites. Porcelain inlays have a higher modulus of elasticity than composites [16].

## Conclusions

When employing inlays and onlays for minimally invasive dentistry, the patient's situation must be carefully selected and evaluated. After that, the dentist should examine the tooth that is being worked on. In order to create a solid bond, the margins have to be situated above the gingiva. Cavities should not extend below the cementoenamel junction. A powerful repair is made possible by preparatory design which decreases physical stress. Smooth flowing margins on the preparatory design are essential for easy repair fabrication. Dentists may be relying too little on the outstanding restorative properties of inlays and onlays. Even though this procedure requires a lot of visits from the patient and assistance in the lab, the results are stable and last for a long time. 
